# Design Optimization of Bulk Piezoelectric Acceleration Sensor for Enhanced Performance

**DOI:** 10.3390/s19153360

**Published:** 2019-07-31

**Authors:** Min-Ku Lee, Seung-Ho Han, Kyu-Hyun Park, Jin-Ju Park, Whung-Whoe Kim, Won-Ju Hwang, Gyoung-Ja Lee

**Affiliations:** 1 Sensor System Research Team, Korea Atomic Energy Research Institute, Daejeon 34057, Korea; 2 #301, 8, Suseong-ro, Gwonseon-gu, Suwon-si, Gyeonggi-do 16426, Korea

**Keywords:** pieoelectric sensor, accelerometer, design, finite-element method, piezoelectric analysis, metamodel

## Abstract

While seeking to achieve high performances of a bulk piezoelectric acceleration sensor, we investigated the behavior of the design variables of the sensor components and optimized the sensor design using a numerical simulation based on piezoelectric analysis and metamodeling. The optimized results demonstrated that there was an exponential dependency in the trade-off relation between two performance indicators, the electric voltage and the resonant frequency, as induced by the design characteristics of the sensor. Among the design variables, a decrease in the base height and epoxy thickness and an increase in the piezo element’s inner diameter had a positive effect on two performances, while the head dimensions (diameter and height) exhibited the opposite effect on them. The optimal sensor designs are proposed within the valid range of resonant frequency (25–47.5 kHz). Our redesign of a commercial reference sensor improved the resonant frequency by 13.2% and the electric voltage by 46.1%.

## 1. Introduction

Pb(Zr,Ti)O_3_ (PZT)-based piezoelectric sensors are used in a wide range of applications in areas of condition and safety monitoring for various industrial facilities owing to their outstanding characteristics such as a fast response, excellent dynamic range and linearity, long term stability and minimal power consumption. Depending on the physical quantity to be measured, both the acoustic emission (AE) sensor and the accelerometer are dominantly used: the AE method is used for analyzing transient elastic waves that come from the elastic deformation energy stored in a solid under stress [1,2,3], while the accelerometer measures the acceleration during oscillations and vibrations on machines and structures [4,5,6].

Ideal sensors for obtaining the best outcomes are sensors that offer, at the same time, a high sensitivity (low threshold) and a high resonant frequency (large frequency usable range) with a small mass (minimal mass loading on the measured object). However, all acceleration sensors are a compromise in functionalities because the main performance indicators of sensitivity and resonant frequency are inextricably linked to each other: the trade-off relation between them is well-known [7,8,9,10,11,12]. More importantly, their performance is totally influenced by the design or structural characteristics of the piezo element and constituent components. Accordingly, the geometry, dimensions and configuration of internal components (e.g., seismic mass, piezo element, tail, base and insulating layer) need to be carefully chosen based on complete understanding of the relationship between the design characteristic and the physical functionality.

Meanwhile, there are always key challenges associated with optimizing the sensor design with respect to the performance. This is because of the existence of a number of alternative designs or configurations of the sensor components, which often make it impossible to test each single configuration by real experiments. The difficulties owing to the high cost and time involved in design optimization via real experiments can be substantially mitigated by the use of numerical simulation. Specifically, establishing analytical models to mimic the geometric design and physical functionality can provide an effective solution for improved design or redesign of the sensor components, which will make it possible to achieve better performance [13,14,15]. Numerical simulation also enables visualization of some details that are difficult to capture in experiments. Consequently, simulation using suitable analytical models can clarify the role of the design variables as well as their connection with the performance behavior. In spite of its strategic importance in developing sensors, numerical studies dealing with the design and control of constituent components of bulk piezoelectric accelerometers to enhance performances appear to be rare.

Using numerical simulations via piezoelectric analysis and metamodeling, in this study we discovered the fundamental behavior of two performance indicators (sensitivity and resonant frequency) as well as their dependence on the design variables of the sensor components. This paper presents our optimum designs of a bulk piezoelectric acceleration sensor with improved sensitivity and resonant frequency. While other piezoelectric transduction modes, such as shear and flexural modes, may be considered, we focused on the compression mode that is in common use. Three-dimensional finite-element modeling was carried out for the analysis and design of the piezoelectric elements and sensor components [16,17,18,19]. A commercial compression-type accelerometer, made of Pb(Zr,Ti)O_3_ (PZT) ceramics, was employed as a design reference for the numerical analysis.

## 2. Materials and Methods

**Figure 1:**
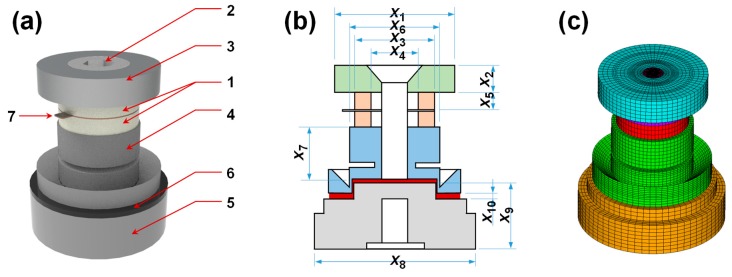
(**a**) Three-dimensional image of compression-type piezoelectric accelerometer module and constituent components (1: Piezo element; 2: Bolt; 3: Head (seismic mass); 4: Tail; 5: Base; 6: Insulating layer; 7: Electrode); (**b**) Ten design variables (*x*_1_ to *x*_10_) used for numerical analysis; (**c**) Finite element model of compression-type accelerometer.

Figure 1a illustrates an elementary design describing a compression-type piezoelectric acceleration sensor with a central preload. The piezo element (1) consists of two ring-type piezoelectric plates cut for the longitudinal effect and oriented with their polarities opposite from the central electrode. They are preloaded by a bolt (2) which acts as a very rigid spring between the head (seismic mass) (3) and the tail (4) and the base (5) plates. The insulating epoxy layer (6) was inserted into the gap between the tail and the base plates. The electrode (7) captures the output signal and feeds it to the connector. When the base plate is accelerated, the seismic mass exerts a proportional force on the piezo element.

Table 1 presents the component materials for the theoretical modeling together with their mechanical properties (density, Young’s modulus and Poisson’s ratio). The head, tail, base and insulating layer materials were tungsten, 316 stainless steel and epoxy, respectively, which are also in common use for the commercial accelerometer. Table 2 presents the property data of PZT ceramic as the piezo element used in this study. All the material property data were used for numerical analysis and modeling.

The standards for the geometry and dimensions of the internal sensor components came from the commercial compression-type piezoelectric accelerometer (CMSS 2106; SKF; Lulea, Sweden) which served as the reference sensor in this work. Ten design variables (*x*_1_ to *x*_10_) and their investigated size ranges were determined based on the reference sensor, as presented in Figure 1b and Table 3.

The finite element analysis (FEA) was performed using a commercial finite element program (ANSYS V18). The total number of elements was 124,800 and the number of nodes was 134,977 (Figure 1c).

Design of experiments (DOE) techniques [20,21] were used to carry out simulation experiments efficiently. The values of the resonant frequency and electric voltage were numerically calculated by piezoelectric analysis using the DOE test points. The total number of DOE test points was 201.

Using the DOE test points, we carried out metamodeling, which is known as more efficient than the actual analytical model [22,23], to approximate the relationship between the design variables (input) and the performances (output). Since the sensor in this work includes many geometrical design variables and nonlinear characteristics, the kriging model [24], which is a global approximation model, was adopted as a metamodel.

Using the kriging model, ten design variables were optimized by a program called Easy Design. The optimization was conducted to find the optimal values of the design variables (*x*_1_ to *x*_10_) by which the electric voltage could be maximized at different resonant frequencies ranging from 20 to 50 kHz. Here, the electric voltage was obtained using a sine-wave acceleration of 1 g at 159 Hz, representing the voltage sensitivity. The optimization process was iterated until a satisfactory result was produced.

For the validation of numerically analyzed results, both numerically simulated and experimentally measured impedance characteristics were obtained and compared. For a real experiment, acceleration sensor modules were fabricated by assembling internal sensor components and piezo elements manufactured with the same materials as those used for the numerical analysis. The sensor components such as head, tail and base were manufactured using the CNC (computer numerically controlled) milling machine tool. The gap between the tail and base was filled with the epoxy (ECCOBOND A 359 LV; Emerson & Curming, Germantown, WI, USA) for insulation. Their designs and dimensions were determined based on the optimization results. The impedance spectra were then measured using an impedance analyzer (SI 1260; Solartron Analytical, Hampshire, UK).

## 3. Results

**Figure 2:**
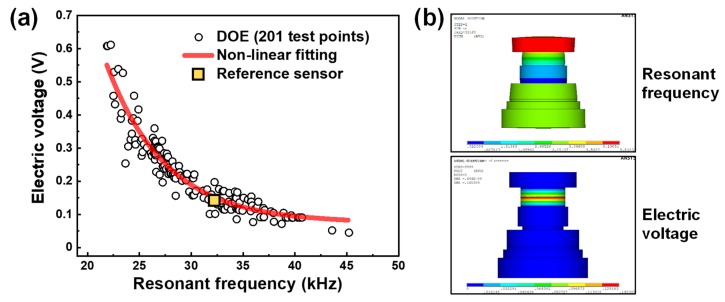
(**a**) Plot for electric voltage vs. resonant frequency obtained from 201 test data by design of experiments (DOE); (**b**) Simulation results of resonant frequency and electric voltage for reference sensor design.

The values of the electric voltage and resonant frequency, obtained from the numerical analysis of 201 test points, are displayed in Figure 2a. The scatterplot, obtained by plotting the electric voltage against the resonant frequency, clearly indicates that their correlation is negative; higher levels of electric voltage are associated with lower levels of resonant frequency. Further, such a negative correlation is nonlinear, even factoring in scattered data. An exponential function fitting is well established, as drawn by a solid red line. The quality of the fit in an exponential negative regression was assessed by determining the coefficient of determination (COD) or *R*^2^ and it was estimated to be 0.9169: an *R*^2^ of 1 indicates that the regression predictions perfectly fit the data.

The trade-off relation between the two performances has been generally accepted in previous studies [8,9,10,11]. The simple equations describing the sensitivity and the resonant frequency are also found elsewhere [25,26]. By considering the pizoelement and seismic mass, the equations for the voltage sensitivity *S_v_* and resonant frequency *f_n_* of the piezoelectric accelerometer are simplified and given by Equations (1) and (2).

(1) $$S_{v}=\frac{4\cdot d_{33}\cdot m_{s}\cdot t_{p}\cdot g}{\varepsilon \cdot \pi \cdot D_{p}{}^{2}}$$

(2) $$\;f_{n}=\frac{1}{2\pi }\cdot {\left(\frac{k}{m_{s}}\right)}^{\frac{1}{2}}=\frac{1}{2\pi }\cdot {\left(\frac{E_{33}\cdot \pi \cdot D_{p}{}^{2}}{4\cdot n\cdot m_{s}\cdot t_{p}}\right)}^{\frac{1}{2}}$$

Here, *n*, *m_s_*, *D_p_*, *t_p_*, *k* and *E_33_* are the number of piezoceramic layer, weight of seismic mass, diameter, thickness, stiffness and Young’s modulus of piezoceramic, respectively. Hence, the *n*, *m_s_*, *D_p_* and *t_p_* are related to the design of the accelerometer, while the *k*, *E_33_* and *d_33_* are related to the material property. From Equations (1) and (2), it can be known that there is a trade-off relation between the sensitivity and resonant frequency: the design variables can only improve one of the performances but with sacrifice to the other. However, none of them reported a negative exponential dependency by considering the whole constituent components (head, piezoelement, tail, base and epoxy) as well as their design variables. Such a negative exponential relation between them provides evidence of association, not causation, as induced by the design characteristics of the sensor.

The scattered data with the exponential dependency in Figure 2a revealed that the degree of scattering of the electric voltage becomes larger as the resonant frequency becomes lower, while the degree of scattering of the resonant frequency is larger at lower electric voltages. Hence, the sensor structure can be designed according to the usage objective and desired performances; for example, a design with either high sensitivity in the low-frequency regime or high resonant frequency for a broad spectrum of sensing can be chosen depending on the requirements.

According to the piezoelectric analysis, the values of resonant frequency and electric voltage obtained from the design of the reference sensor were 32.2 kHz and 0.145 V, respectively (Figure 2b). It can be seen that such values exist on the line of the fitting curve, as indicated by the yellow square in Figure 2a.

**Figure 3:**
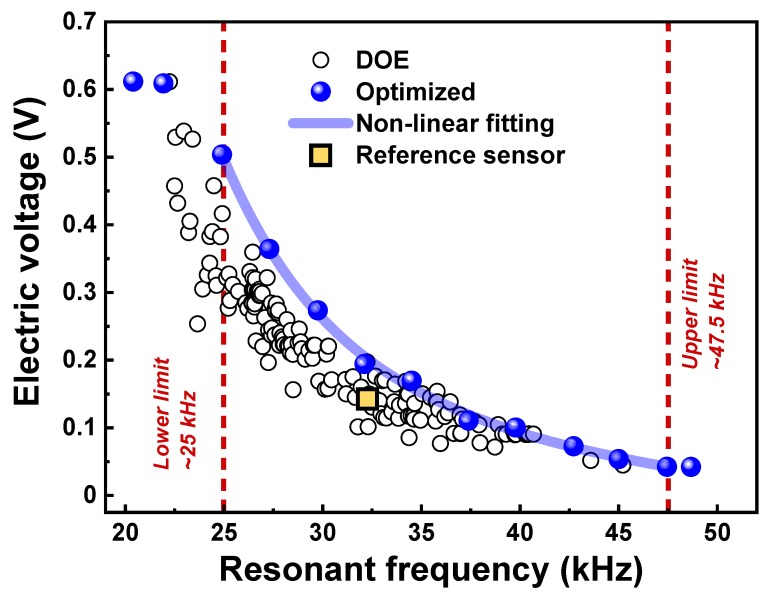
Plot for electric voltage vs. resonant frequency obtained through optimization process at different resonant frequencies (20–50 kHz).

Based on the metamodel obtained from a number of DOE test points, the optimization was carried out with respect to target resonant frequencies ranging from 20 to 50 kHz. The optimized results of ten design variables and the corresponding values of resonant frequency and electric voltage are presented in Figure 3 and Table 4. Firstly, we noted that the model calculation for optimization failed to converge at target resonant frequencies of 20, 22 and 50 kHz owing to the dimensional limits of size range of the design variables, as shown in Table 4. Hence, the valid range of the resonant frequency for the metamodel was determined to be between 25–47.5 kHz. In this range, there was a strong negative exponential relationship with an excellent COD (or *R*^2^) value of 0.99819, which was close to 1, as indicated by the solid blue line in Figure 3. We concluded that the highest values of resonant frequency and electric voltage, obtainable from the presented sensor geometry and range of component dimensions, exist on this blue line.

**Figure 4:**
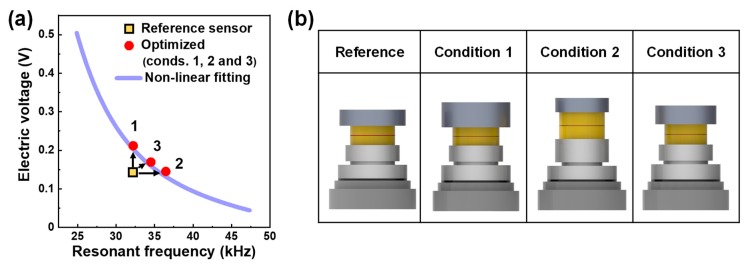
(**a**) Plot for electric voltage vs. resonant frequency obtained after optimizing at conditions 1, 2 and 3; (**b**) Images of corresponding optimized designs.

Next, the optimization was further carried out to determine how much the performance can be increased compared to that obtained from the reference sensor and what design variables influence the resulting behavior. For this, three different conditions were adopted, referred to as conditions 1, 2 and 3. For condition 1, the electric voltage was optimized at a constant resonant frequency (32.2 kHz), while, for condition 2, the resonant frequency was optimized at a constant electric voltage (0.145 V). Finally, condition 3 was made to optimize both performances. The results after optimizing for the three conditions are plotted by the red circles in Figure 4a. It was clear that such optimizations were successful in improving one of the performances (conditions 1 and 2) or both of them (condition 3).

The optimized values of the electric voltage and resonant frequency were also placed on the line of the nonlinear fitting curve, as already shown in Figure 3. For condition 1, the electric voltage increased to 0.212 V with a 46.1% increase, while, for condition 2, the resonant frequency increased to 36.457 kHz with a 13.2% increase. For condition 3, the electric voltage increased by 17.7% (0.170 V) and the resonant frequency by 7.2% (34.515 kHz). As shown in Figure 4b, the corresponding cross-sectional sensor images could be drawn using the values of the design variables optimized at each condition (Table 5). The optimized designs showed clear differences from that of the reference sensor: note the piezo elements and constituent components of different sizes.

**Figure 5:**
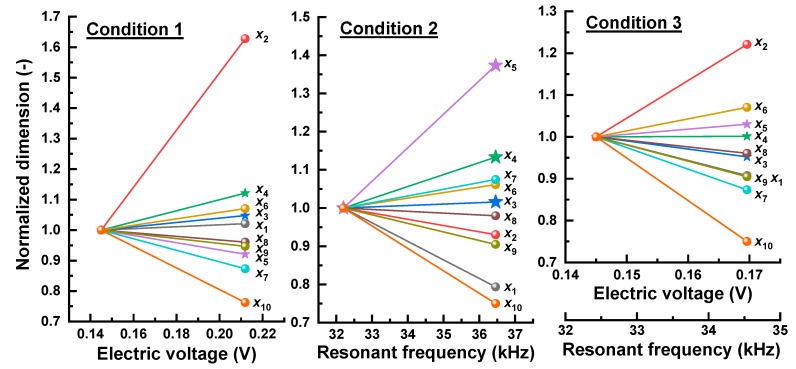
Variation of normalized dimension of each design variable with respect to reference design for conditions 1, 2 and 3.

To investigate the effects of the design variables on the observed behavior in detail, the variation of ten design variables are presented in Figure 5 for the three conditions with the dimensions normalized with respect to those of the reference sensor. For condition 1, the design variables mainly affecting the increase in electric voltage were *x*_2_ (head height), *x*_4_ (piezo element I.D.), *x*_7_ (tail height), *x*_9_ (base height), and *x*_10_ (epoxy thickness). The head height and piezo element I.D. should be increased by 63% and 12%, respectively, whereas the tail height and epoxy thickness should be decreased by 13% and 24%, respectively. The impacts of the remaining design variables were insignificant (less than 10%). As for condition 2, the increased resonant frequency was mainly induced by *x*_1_ (head O.D.), *x*_4_ (piezo element I.D.), *x*_5_ (piezo element thickness), *x*_7_ (tail height), *x*_9_ (base height) and *x*_10_ (epoxy thickness). For instance, the piezo element I.D. and piezo element thickness should be increased by 13% and 37%, respectively. On the other hand, the head O.D., base height and epoxy thickness should be decreased by 21%, 10% and 25%, respectively. For condition 3, both performances could be increased due to a 10% decrease in *x*_1_ (head O.D.), 22% increase in *x*_2_ (head height), 13% decrease in *x*_7_ (tail height), 10% decrease in *x*_9_ (base height) and 25% decrease in *x*_10_ (epoxy thickness).

As a result, the decrease in base height and epoxy thickness as well as the increase in piezo element I.D. had a positive effect on both performances. Since the data in Figure 5 on the other design variables (head height, piezo element thickness and tail height) show that one performance has a positive effect and one a negative effect, those variables need to be properly controlled by the selection of an increase or a decrease to meet the performance requirements. Notably, the head dimensions (O.D. and height) were the most influential factor and exhibited the opposite effect on the performances. This is evidence for the conflicting actions of seismic mass between the resonant frequency and sensitivity. The volume-to-weight conversion indicated that the head weight should be decreased by 42% to enhance the resonant frequency for condition 2, whereas it should be increased by 69% to enhance the electric voltage for condition 1. Interestingly, the balanced improvement of the two performances, obtained from condition 3, could be achieved only by the dimensional changes of the head (O.D. and height) with maintaining the total weight. These results suggest that it is important to determine the optimal dimensions of the head.

**Figure 6:**
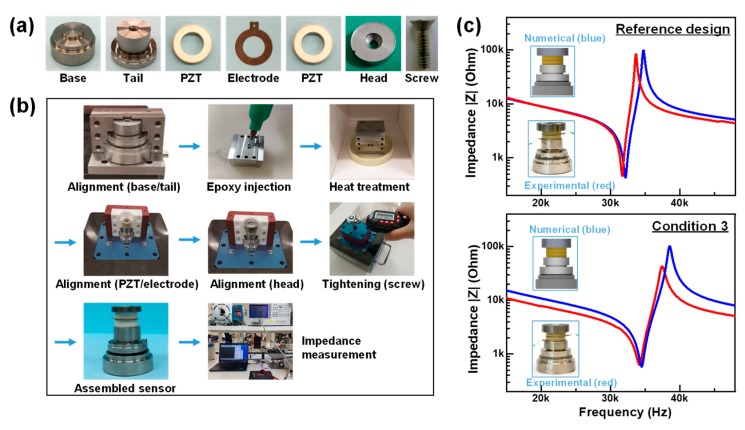
(**a**) Images of internal components fabricated according to the optimized design (condition 3 in Table 5); (**b**) Fabrication procedure of a PZT acceleration sensor module used for measurement of impedance characteristics; (**c**) Impedance spectra obtained from sensor modules (reference design and condition 3). The blue and red lines indicate the numerically simulated and experimentally measured impedance curves, respectively.

To evaluate the reliability of the numerically analyzed results, the impedance characteristics, related to the resonance characteristics, obtained from the numerical analysis were compared with those experimentally measured for the sensor module fabricated with the same materials and dimensions. Two sensor modules were fabricated using internal components and piezo elements manufactured according to the optimized design of condition 3 (Figure 6a) and the reference sensor design. The typical fabrication procedure showing assembling steps of an acceleration sensor module is illustrated in Figure 6b. Since the alignment of the sensor components are critical to the performances, we used the specially designed zig for highly accurate and reliable assembling of the constituent components. According to the results in Figure 5, moreover, the epoxy thickness was sensitive to the performances. In order to precisely control the amount of injected epoxy, we also used an injection mold of our own design. In tightening with a screw, a torque value was optimized by examining the resonance frequency with the variation of the torque value. As a result, the resonance frequency was found to be stabilized at a torque value of about 2.0 Nm and, accordingly, the aligned sensor components were tightened under a uniform torque of 2.0 Nm using a digital torque wrench.

Finally, both numerically simulated and experimentally measured impedance spectra for the two sensor module designs were obtained, as shown in Figure 6c. The results clearly showed the very similar impedance-frequency characteristic between the experimentally measured and numerically simulated spectra. For the reference sensor, the experimental resonant and antiresonant frequencies were 31.7 and 33.7 kHz, respectively, while the respective values obtained from the numerical analysis were 32.2 and 34.8 kHz. For the optimized design of condition 3, their respective experimental values were 34.1 and 37.4 kHz, which were nearly consistent with those of the numerical analysis (34.5 and 38.5 kHz). The observed consistency between the numerical and experimental resonance characteristics strongly suggests the validity of the numerically analyzed results.

## 4. Conclusions

In this study, using piezoelectric analysis and metamodeling, we investigated the behavior of two performance indicators, electric voltage and resonant frequency, as well as their dependence on the design variables of constituent components in bulk piezoelectric acceleration sensors. The simulated results revealed that there was a negative exponential relationship between the electric voltage and the resonant frequency: *R*^2^ ~ 0.99819 for the optimized data. Concerning the effects of design variables, the decrease in base height and epoxy thickness as well as the increase in piezo element I.D. had a positive effect on both performances, while the head height, piezo element thickness and tail height had a positive effect on one performance and a negative effect on the other performance. The head dimensions (O.D. and height) also confirmed the conflicting actions of seismic mass on the resonant frequency and sensitivity. Based on them, we proposed the optimal sensor designs within the valid resonant frequency range (25–47.5 kHz) for the presented metamodel. We also confirmed that the redesign of the commercial reference sensor based on the optimization successfully improved the performances, i.e., 13.2% (32.2 to 36.457 kHz) for the resonant frequency and 46.1% (0.145 to 0.212 V) for the electric voltage. The validity of the simulated results was proved by nearly consistent impedance characteristics between the numerical and experimental outcomes.

## Figures and Tables

**Table 1:** Component materials and their mechanical properties (density, Young’s modulus, and Poisson’s ratio).

Component	Material	Density (kg/m^3^)	Young’s Modulus (GPa)	Poisson’s Ratio (-)
Head	Tungsten	17,900	385	0.2
Tail	316 stainless steel	7767	193	0.29
Base	316 stainless steel	7767	193	0.29
Insulating layer	Epoxy	1400	9.5	0.35

**Table 2:** Electrical and mechanical properties of Pb(Zr,Ti)O_3_ (PZT).

Physical Properties		Electrical Properties (at 25 ^°^C)			Elastic Compliance						Frequency Constant		
Density (kg/m^3^)	Poisson’s ratio (-)	*d*_31_ (pm/V)	*d*_33_ (pm/V)	*d*_15_ (pm/V)	S_11_^E^	S_12_^E^	S_13_^E^	S_33_^E^	S_44_^E^	S_66_^E^	N_L_	N_T_	N_P_
7600	0.3	−185	392	471	15.64	−5.47	−7.32	19.05	46.02	42.23	1445	2005	1958

**Table 3:** Ten design variables and their size ranges used for numerical modeling.

Design Variables	Description	Reference Dimension (mm)	Size Range (mm)
*x*_1_	Head outer diameter (O.D.)	19	15–23
*x*_2_	Head height	4.3	4–7
*x*_3_	Piezoelement outer diameter (O.D.)	12.6	12–13.2
*x*_4_	Piezoelement inner diameter (I.D.)	7.5	6.5–8.5
*x*_5_	Piezoelement thickness	2.65	1.65–3.65
*x*_6_	Tail outer diameter (O.D.)	14.2	13.2–15.2
*x*_7_	Tail height	7.9	6.9–8.9
*x*_8_	Base outer diameter (O.D.)	25.4	24.4–26.4
*x*_9_	Base height	10.5	9.5–11.5
*x*_10_	Epoxy thickness	0.8	0.6–1.0

**Table 4:** Values of optimized design variables at different target resonant frequencies and corresponding resonant frequency and electric voltage.

Cond. ^1^ (kHz)	*x*_1_ (mm)	*x*_2_ (mm)	*x*_3_ (mm)	*x*_4_ (mm)	*x*_5_ (mm)	*x*_6_ (mm)	*x*_7_ (mm)	*x*_8_ (mm)	*x*_9_ (mm)	*x*_10_ (mm)	R.F (kHz)	E.V. (V)	Remark ^2^
20	23	7	12	8.5	3.65	13.2	6.9	26.4	11.5	1	20.4	0.612	F
22	23	7	12	8.5	3.65	15.2	6.9	26.4	11.5	0.6	21.931	0.609	F
25	22.83	7	12.47	8.48	3.47	15.2	6.93	24.4	11.46	0.6	24.901	0.504	S
27.5	21.61	7	12	7.22	3.03	15.19	6.9	24.81	9.5	0.6	27.301	0.364	S
30	21	7	13.2	8.5	2.65	15.2	6.9	24.4	11.02	0.6	29.758	0.274	S
32.5	16.9	6.36	13.1	6.93	3.43	15.2	8.69	24.74	11	0.63	32.122	0.194	S
35	17.24	5.25	12	7.51	2.73	15.2	6.9	24.4	9.5	0.6	34.515	0.170	S
37.5	15	5.27	12.75	7.5	2.65	15.2	7.91	26.29	10.52	0.6	37.394	0.111	S
40	15	4	13.2	7.76	3.04	15.2	7.53	24.4	11.5	0.76	39.786	0.1	S
42.5	15.62	4.51	12.41	8.5	1.65	15.2	7.31	24.4	9.78	0.6	42.713	0.073	S
45	15	4	12	6.98	1.65	15.2	7.12	25.87	9.5	0.6	44.995	0.054	S
47.5	15	4	13.2	6.5	1.66	14.94	6.9	25	9.53	0.6	47.45	0.424	S
50	15	4	13.2	6.5	1.65	15.2	6.9	24.4	9.5	0.6	48.667	0.422	F

^1^ Conditions of target resonant frequency used for numerical simulation; ^2^ Failure (F) or success (S) of convergence during optimization.

**Table 5:** Values of optimized design variables for conditions 1, 2 and 3 and corresponding values of resonant frequency and electric voltage.

Cond.	*x*_1_ (mm)	*x*_2_ (mm)	*x*_3_ (mm)	*x*_4_ (mm)	*x*_5_ (mm)	*x*_6_ (mm)	*x*_7_ (mm)	*x*_8_ (mm)	*x*_9_ (mm)	*x*_10_ (mm)	R.F. (kHz)	E.V. (V)
1	19.4	7	13.2	8.41	2.44	15.2	6.9	24.4	9.94	0.61	32.212	0.212
2	15.08	4	12.8	8.5	3.64	15.07	8.49	24.89	9.5	0.6	36.457	0.145
3	17.24	5.25	12	7.51	2.73	15.2	6.9	24.4	9.5	0.6	34.515	0.170
Reference	19	4.3	12.6	7.5	2.65	14.2	7.9	25.4	10.5	0.8	32.2	0.145
